# Five‐day water‐only fasting decreased metabolic‐syndrome risk factors and increased anti‐aging biomarkers without toxicity in a clinical trial of normal‐weight individuals

**DOI:** 10.1002/ctm2.502

**Published:** 2021-07-29

**Authors:** Yanyu Jiang, Xi Yang, Changsheng Dong, Yun Lu, Hongmei Yin, Biying Xiao, Xuguang Yang, Wenlian Chen, Wei Cheng, Hechuan Tian, Lin Guo, Xiaobo Hu, Hong Fang, Weiqin Chen, Zhen Li, Wenqin Zhou, Weijun Sun, Xiyan Guo, Shaobin Li, Yuli Lin, Rui He, Xiaoyun Chen, Di Liu, Minghui Zhang, Yanmei Zhang, Hu Zhao, Peiyong Zheng, Thomas N. Seyfried, Robert M. Hoffman, Wei Jia, Guang Ji, Lijun Jia

**Affiliations:** ^1^ Cancer Institute Longhua Hospital Shanghai University of Traditional Chinese Medicine Shanghai China; ^2^ Department of Clinical Laboratory Longhua Hospital Shanghai University of Traditional Chinese Shanghai China; ^3^ Preventive Care Center of TCM Longhua Hospital Shanghai University of Traditional Chinese Medcine Shanghai China; ^4^ Nursing Department Longhua Hospital Shanghai University of Traditional Chinese Medicine Shanghai China; ^5^ Department of Immunology School of Basic Medical Sciences Fudan University Shanghai China; ^6^ Department of Rheumatology and Immunology Longhua Hospital Shanghai University of Traditional Chinese Medicine Shanghai China; ^7^ Computational Virology Group Center for Bacteria and Viruses Resources and Bioinformation Wuhan Institute of Virology Chinese Academy of Sciences Beijing China; ^8^ School of Medicine of Tsinghua University Beijing China; ^9^ Department of Laboratory Medicine Huadong Hospital Fudan University Shanghai China; ^10^ Institute of Digestive Diseases Longhua Hospital Shanghai University of Traditional Chinese Medicine Shanghai China; ^11^ Biology Department Boston College Chestnut Hill Massachusetts USA; ^12^ Department of Surgery University of California San Diego California USA; ^13^ AntiCancer Inc San Diego California USA; ^14^ University of Hawaii Cancer Center Honolulu Hawaii USA

Dear Editor,

Fasting is known to have many health benefits such as prolonging lifespan and suppression of tumorigenesis.^1^, ^2^, ^3^ In the present study, we systematically evaluated the effects of water‐only fasting on metabolic‐syndrome and age‐related risk markers in 45 normal‐weight individuals.

As shown, a 4.59 kg reduction in body weight, 9.85 cm reduction in waist circumference, and 1.64 kg/m^2^ reduction in body mass index (BMI) were observed during a 5‐day water‐only fast (Figures 1A‐1C). After refeeding for 1 month (day 38), body weight, waist circumference, and BMI were still lower than the baseline level (Figures 1A‐1C). Blood pressure (BP) significantly declined during water‐only fasting with diastolic BP declining more than systolic BP and gradually both increased to the baseline level by 98 d (Figures 1D and 1E). Considering many fasting studies showed diastolic BP reduction did not exceed systolic BP reduction, future studies are needed on water‐only fasting and BP reduction. Insulin dropped approximately 2.8‐fold lower than the baseline level during water‐only fasting (Figure 1F). Insulin‐like growth factor 1 (IGF‐1) decreased by a total of 26% during water‐only fasting and decreased more in females than males (Figure 1G and Table S1). Future studies will address the sexual disparity of IGF‐1 reduction during water‐only fasting. The number of pan T cells, CD4+T cells, CD8+T cells, and B cells decreased during water‐only fasting (Figures 1H‐1K). In contrast, the frequency of Treg cells significantly increased during fasting and still exceeded the baseline level 3 months after refeeding (Figures 1L and 1M). This is an important benefit, since Treg cells have anti‐inflammation effects.^4^ With regard to thyroid hormones, T4 increased rapidly during fasting, whereas T3 and TSH decreased (Figures 1N‐1P). The decreased level of T3 during water‐only fasting is of particularly importance since a low T3 level, without impairing thyroid function, is strongly associated with longevity.^5^, ^6^ The present study suggested that water‐only fasting for many parameters was similar to calorie restriction and a fasting‐mimic diet.^6^, ^7^, ^8^, ^9^

**FIGURE 1:**
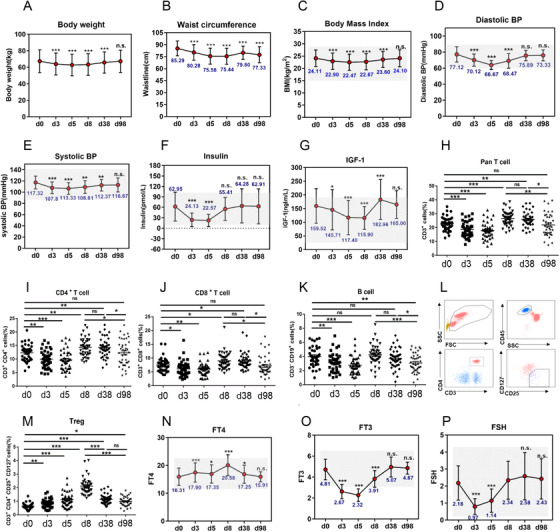
Water‐only fasting reduced metabolic‐syndrome and aging risk markers. (A‐G) Effects of fasting on the metabolic syndrome‐related bio‐markers, bodyweight (A), waist circumference (B), body mass index (C), systolic blood pressure (D), diastolic blood pressure (E), insulin (F), insulin‐like growth factor 1 (IGF‐1) (G). Forty‐one participants (17 male and 24 female) completed this clinical trial, and all participants who provided serum and urine samples at baseline (Day 0), fasting period (day 3, day 5), 3 days (day 8), and 1 month post‐water‐only fasting (day 38), were included. (H‐M) Dynamics of the frequency of immune populations were detected upon water‐only fasting. Fasting peripheral venous blood samples were collected by trained medical‐technical personnel. (O) Regulatory T cells were identified as CD45+CD3+CD4+CD25+CD127‐cells. Representative FCM data are shown (L). (M) Dynamics of the frequency of Tregs at indicated time‐points in water‐only fasting or refeeding procedure. (N‐P) The levels of free thyroxine (T4), triiodothyronine(T3), and thyroid‐stimulating hormone (TSH) were determined during fasting and refeeding. n.s denotes not significant, *p < 0.05, **p < 0.01,***p < 0.001

Metabolomic profiling of serum and urine was preformed to investigate the underlying metabolic mechanisms of water‐only fasting. Principal‐component analysis (PCA), volcano‐plots, and heatmaps illustrated that the level of many metabolites during water‐only fasting differed from those at baseline (Figures 2A‐2D and Table S2). KEGG metabolic‐pathway analyses showed that water‐only fasting significantly impacted five metabolic pathways in serum, including synthesis and degradation of ketone bodies (Figure 2E). In urine, ketone bodies and TCA cycle metabolites were significantly altered (Figure 2F). In the glucose‐metabolism pathway, glucose, pyruvate, and lactate decreased, whereas isocitric acid and malic acid increased in serum. Citric acid decreased in serum and increased in urine. After 1 month refeeding, all these five metabolites, except for lactic acid, returned to the baseline level (Figure 2G   and S2). These results show that glycolysis was inhibited during water‐only fasting.

**FIGURE 2:**
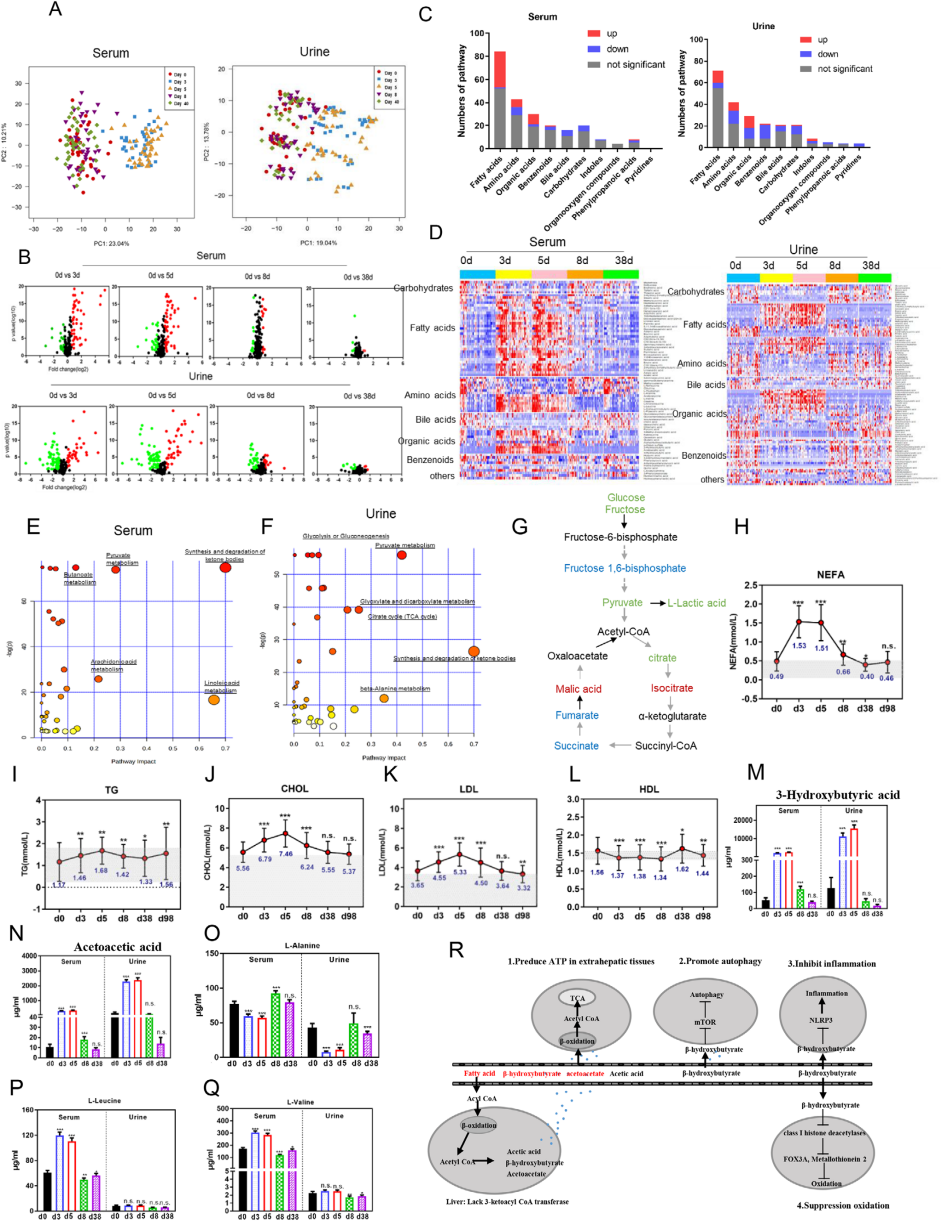
Metabolic reprogramming during water‐only fasting. (A) Principal‐component analysis (PCA) in serum and urine. (B) Volcano plot analyses in serum and urine using all metabolites. Forty‐one participants (17 male and 24 female) completed this clinical trial, and all participants who provided serum and urine samples at baseline (Day 0), fasting period (day 3, day 5), 3 days (day 8), and 1 month post‐fasting (day 38) were included in metabolomic sequencing and analysis. (C and D) Histogram and heatmap showing the changes of fatty acids and organic acids, carbohydrates, and bile acids in serum and in urine after a 5‐day water‐only fast. (E and F) KEGG metabolic pathway analyses in serum and urine. Altered metabolites were selected and metabolic pathways analyzed and described in the Metabo analyst website (https://www.metaboanalyst.ca/MetaboAnalyst/docs/RTutorial.xhtml). (G) A total of six metabolites involved in glucose metabolism were differentially expressed in both serum and urine, including glucose, pyruvate acid, isocitric acid, malic acid, citric acid, and lactate acid. Green denotes downregulated; red denotes upregulated; and blue denotes not changed. Black denotes not detected. (H‐N) The effect of water‐only fasting on fuel‐source markers, including: nonesterified fatty acid (NEFA), triglyceride (TG), cholesterol (CHOL), low‐density lipoprotein (LDL), high‐density lipoprotein (HDL), acetoacetate, β‐hydroxybutyrate. (O‐Q) The effects of water‐only fasting on amino acids (valine, leucine, therosine, alanine). n.s denotes not significant, *p < 0.05, **p < 0.01, ***p < 0.001

Further analysis showed that three types of lipids, including triglycerides, cholesterol, and non‐esterified fatty acids, significantly increased during water‐only fasting period (Figures 2H‐2J). Low‐density lipoprotein (LDL) increased, whereas high‐DL (HDL) decreased during water‐only fasting (Figures 2K and 2L). All these five indexes gradually returned to the basal level after refeeding. These unfavorable changes caution that 1) water‐only fasting might be not suitable for dyslipidemia patients; 2) lipid levels should be closely monitored during water‐only fasting. High levels of fatty acids, which are released from adipose and other tissues during fasting, are oxidized for energy production. Whereas the liver only partially oxidizes fatty acids into ketone bodies, which are then released into the blood and used extrahepatically to produce energy, especially for the brain. Consistently, the percentage of ketone bodies increased by 84.4% in urine above grade 3 after 3 days fasting and 95.2% after 5 days fasting, whereas all decreased below grade 3 after refeeding (Table 1). Among the ketone bodies, β‐hydroxybutyrate increased as much as 51‐fold (Figure 2M), and acetoacetate increased up to 31‐fold during fasting (Figure 2N). The high level of β‐hydroxybutyrate during 5‐day water‐only fasting is of particularly importance as β‐hydroxybutyrate is a specific inhibitor of class I histone deacetylases which results in protection against oxidative stress and is an important anti‐aging factor^10^ (Figure 2R). Such large increase in ketone bodies such as β‐hydroxybutyrate are more extensive during water‐only fasting compared to partial calorie restriction and fasting mimicking diet.^2^, ^6^, ^7^, ^8^, ^9^ Furthermore, serum levels of circulating branch‐chain amino acids (valine and leucine) significantly increased, whereas alanine, the major gluconeogenic amino acid, decreased upon fasting (Figures 2O‐2Q). The results demonstrate that the cellular fuel source changed from glucose to ketone bodies, lipids and proteins to adapt to water‐only fasting, indicating metabolic flexibility of the human body to match the fasting‐induced energy demand to maintain vital physical functions.

**TABLE 1:** Ketone bodies increased during water‐only fasting

Ketone body	D0 (%)	D3 (%)	D5 (%)	D8 (%)	D38 (%)	D98 (%)
Grade 0	32 (78.1)	1 (2.4)	0 (0)	26 (63.4)	41 (100)	39 (95.1)
Grade 1	7 (17.1)	0 (0)	1 (2.4)	13 (31.7)	0 (0)	0 (0)
Grade 2	1 (2.4)	1 (2.4)	1 (2.4)	2 (4.9)	0 (0)	2 (4.9)
Grade 3	1 (2.4)	10 (24.4)	2 (4.9)	0 (0)	0 (0)	0 (0)
Grade 4	0 (0)	29 (70.8)	37 (90.3)	0 (0)	0 (0)	0 (0)
Z		−5.647	−5.837	−0.965	−2.807	−1.589
P		<0.001	<0.001	0.335	0.005	0.112

Safety‐associated indexes, including liver function, renal function, plasma electrolytes, and blood‐cell count fluctuated within the normal ranges, except for uric acid, during water‐only fasting (Table 2), indicating that water‐only fasting might not be suitable for gout patients and patients with impaired renal function. Fasting enhanced the feeling of hunger by approximately 4‐fold to a moderate degree after fasting for 5 days than the baseline level (Figure S1A). Psychological indexes including scales of anxiety (Hamilton Anxiety, HAMA) and depression (Hamilton Depression, HAMD) significantly decreased on average by 1.32 and 0.81 points during the clinical trial (Figure S1B), which may have been influence by meditation. No adverse events including diarrhea, cramps, nausea, fatigue, headache, dizziness, fever, stomach ache, insomnia were reported by the participants and our clinical team (data not shown). These findings collectively suggest that 5‐day water‐only fasting was safe for healthy subjects.

**TABLE 2:** Effects of water‐only fasting on liver function, renal function, plasma electrolytes, and blood‐cell counts

		Mean ± SD						*p* value			Changes			
		0d	3d	5d	8d	38d	98d	0d vs. 5d	0d vs. 38d	0d vs. 98d	∆5d–0d	∆38d–0d	∆98d–0d	Reference range
Liver function	ALT	20.20 ± 13.82	16.56 ± 10.31	15.58 ± 7.78	15.58 ± 6.68	17.72 ± 9.97	15.20 ± 9.74	0.00689	0.18841	0.02371	−4.62	−2.48	−5	7–45
	AST	23.30 ± 15.73	22.82 ± 7.44	24.04 ± 8.01	22.93 ± 7.32	20.30 ± 5.97	20.43 ± 4.96	0.76919	0.20843	0.25455	0.74	−3	−2.87	13–40
	GGT	23.54 ± 14.99	22.66 ± 13.10	22.31 ± 12.52	19.52 ± 9.70	21.13 ± 11.87	18.42 ± 12.48	0.02829	0.04005	0.00029	−1.23	−2.41	−5.12	7–45
	TP	74.25 ± 3.97	77.54 ± 3.70	80.14 ± 4.22	75.47 ± 4.37	73.14 ± 3.51	73.86 ± 3.54	0.00000	0.07762	0.53148	5.89	−1.11	−0.39	65–85
	ALB	46.02 ± 2.69	49.00 ± 2.47	50.18 ± 2.57	47.04 ± 3.13	45.23 ± 2.83	45.42 ± 2.77	0.00000	0.03758	0.12334	4.16	−0.79	−0.6	40–55
	PALB	0.31 ± 0.06	0.27 ± 0.06	0.24 ± 0.05	0.29 ± 0.05	0.31 ± 0.05	0.32 ± 0.06	0.00000	0.89447	0.06379	−0.07	0	0.01	0.25–0.40
Renal function	TBA	4.36 ± 4.30	1.88 ± 3.36	1.88 ± 2.85	3.35 ± 4.57	4.00 ± 2.81	3.60 ± 2.67	0.00101	0.57131	0.31397	−2.48	−0.36	−0.76	0.10–10
	CG	1.71 ± 0.82	1.35 ± 1.92	1.20 ± 1.15	1.59 ± 2.60	1.59 ± 0.54	1.61 ± 0.54	0.00070	0.16492	0.34285	−0.51	−0.12	−0.1	0.10–2.70
	UA	365.80 ± 107.94	627.07 ± 121.27	787.85 ± 142.28	452.20 ± 160.82	347.73 ± 102.05	332.39 ± 117.77	0.00000	0.01767	0.01234	422.05	−18.07	−33.41	155–357
	CREA	62.30 ± 12.74	68.00 ± 12.60	70.61 ± 14.10	69.38 ± 14.64	64.18 ± 13.13	63.02 ± 12.68	0.00000	0.04866	0.51724	8.31	1.88	0.72	41–81
	BUN	6.06 ± 1.55	6.85 ± 1.82	5.82 ± 1.65	3.70 ± 1.52	4.91 ± 1.02	4.97 ± 1.34	0.31795	0.00003	0.00003	−0.24	−1.15	−1.09	2.86–7.14
Plasma electrolytes	Na	141.16 ± 1.44	139.44 ± 1.72	138.69 ± 1.71	142.05 ± 1.76	141.73 ± 1.79	141.48 ± 1.33	0.00000	0.01198	0.10420	−2.47	0.57	0.32	137–147
	Cl	103.36 ± 1.52	99.72 ± 1.78	99.05 ± 2.42	103.18 ± 2.35	104.72 ± 2.06	104.08 ± 2.07	0.00000	0.00003	0.03100	−4.31	1.36	0.72	96–108
	K	4.28 ± 0.27	4.46 ± 0.28	4.40 ± 0.34	4.00 ± 0.75	4.29 ± 0.31	4.21 ± 0.26	0.07598	0.98684	0.15999	0.12	0.01	−0.07	3.50–5.30
	Ca	2.42 ± 0.06	2.47 ± 0.09	2.48 ± 0.09	2.52 ± 0.09	2.39 ± 0.09	2.40 ± 0.10	0.00002	0.02916	0.16725	0.06	−0.03	−0.02	2.11–2.52
	Mg	0.89 ± 0.05	0.91 ± 0.07	0.91 ± 0.06	0.89 ± 0.06	0.93 ± 0.05	0.95 ± 0.06	0.00738	0.00000	0.00000	0.02	0.04	0.06	0.75–1.02
Blood routine	WBC	5.77 ± 1.68	5.68 ± 1.51	5.80 ± 1.67	4.64 ± 1.19	5.48 ± 1.37	5.56 ± 1.52	0.90550	0.09298	0.38415	0.03	−0.29	−0.21	3.50–9.50
	Lymphocytes	1.87 ± 0.53	1.44 ± 0.46	1.74 ± 0.52	1.85 ± 0.51	1.90 ± 0.53	1.81 ± 0.57	0.06642	0.54063	0.34875	−0.13	0.03	−0.06	1.10–3.20
	Monocytes	0.40 ± 0.12	0.34 ± 0.13	0.44 ± 0.17	0.38 ± 0.13	0.41 ± 0.13	0.41 ± 0.13	0.13226	0.88017	0.88017	0.04	0.01	0.01	0.10–0.60
	Eosinophil granulocytes	0.11 ± 0.08	0.06 ± 0.06	0.06 ± 0.05	0.09 ± 0.06	0.12 ± 0.08	0.12 ± 0.08	0.00000	0.35960	0.62520	−0.05	0.01	0.01	0.02–0.52
	Neutrophil granulocytes	3.36 ± 1.13	3.81 ± 1.25	3.53 ± 1.21	2.30 ± 0.80	3.01 ± 0.87	3.19 ± 1.07	0.32400	0.00785	0.41451	0.17	−0.35	−0.17	1.80–6.30
	Basophil granulocytes	0.03 ± 0.01	0.03 ± 0.02	0.03 ± 0.02	0.03 ± 0.02	0.03 ± 0.01	0.03 ± 0.01	0.07890	0.36094	0.36094	0	0	0	0.00–0.60
	reticulocytes	70.60 ± 24.39	67.50 ± 23.52	61.31 ± 22.32	37.94 ± 14.53	67.92 ± 19.58	57.43 ± 23.57	0.00022	0.33304	0.00001	−9.29	−2.68	−13.17	17–70.1
	RBC	4.60 ± 0.49	4.88 ± 0.49	5.03 ± 0.51	4.73 ± 0.50	4.48 ± 0.46	4.57 ± 0.45	0.00000	0.01919	0.38884	0.43	−0.12	−0.03	3.80–5.10
	HGB	140.51 ± 17.72	148.24 ± 17.56	153.68 ± 18.55	143.78 ± 16.95	137.95 ± 17.63	141.56 ± 18.30	0.00000	0.09092	0.39672	13.17	−2.56	1.05	115–150
	Platelets	266.95 ± 88.35	282.71 ± 88.14	281.24 ± 93.83	255.96 ± 91.43	265.34 ± 81.91	272.95 ± 86.46	0.02036	0.74669	0.24557	14.29	−1.61	6	125–350

Abbreviations: ALT, alanine aminotransferase; AST, aspartate transerese; GGT, gamma‐glutamyltranst; TP, total protein; ALB, albumin; PALB: prealbumin;TBA, total bile acid; CG, cholyglycine; UA, uric acid; CREA, creatinine; BUN, blood urea nitrogen; Na, sodium; Cl, chlorine; K, kalium; Ca, calcium; Mg, magnesium; WBC, white blood cell; RBC, red blood cell; HGB, hemoglobin.

In summary, the present study suggests that 5‐day water‐only fasting reduces metabolic‐syndrome and aging biomarkers. Water‐only fasting upregulates Tregs to prevent or treat inflammation‐related diseases, as well as potentially promote anti‐aging by decreasing T3, insulin, IGF‐1, and significantly increasing β‐hydroxybutyrate. The results of the present study are very promising as 5‐day water‐only fasting has many critical beneficial effects without toxicity. Because the present trial is carried out in specialized clinics, water‐only fasting should be guided by clinical team and may not be applicable to general populations. Furthermore, participants who follow healthy diet may have better long‐term outcomes than participants with unhealthy diet. A future water‐only fasting clinical trial will test the efficacy on obese patients.

## CONFLICT OF INTEREST

The authors declare that they have no conflict of interest.

## FUNDING INFORMATION

This work was supported by National Natural Science Foundation of China (grant numbers: 81625018, 81820108022, 82003297 and 81620108030), Innovation Program of Shanghai Municipal Education Commission (2019‐01‐07‐00‐10‐E00056), Program of Shanghai Academic/Technology Research Leader (18XD1403800), National 13th 5‐Year Science and Technology Major Special Project for New Drug and Development (2017ZX09304001), ChenGuang project supported by Shanghai Municipal Education Commission and Shanghai Education Development Foundation (19CG49).

## Supporting information

Supporting information
