# Supplementary material for: Five‐day water‐only fasting decreased metabolic‐syndrome risk factors and increased anti‐aging biomarkers without toxicity in a clinical trial of normal‐weight individuals
Source: Clin Transl Med. 2021 Jul 29;11(8):e502. doi: 10.1002/ctm2.502 (PMC8320652; doi:10.1002/ctm2.502)
Supplement: Supplementary file 1 — Supporting information [file CTM2-11-e502-s001.doc]

**Supplementary materials**

# Materials and Methods

**Study oversight**

Forty-five volunteers from six provinces of China were recruited and 41 of the volunteers completed the water-only fasting (also known as Bigu, a traditional Chinese fasting mode) clinical trial. The subjects were healthy and within the normal weight range (Table S3). The present trial was conducted at Longhua Hospital, Shanghai University of Traditional Chinese Medicine. Each participant gave written informed consent. The protocol was approved by the Ethics Institutional Review Board of Shanghai University of Traditional Chinese Medicine (2017LCSY049) and registered at ClinicalTrials.gov (NCT03754920).

**Study design**

Inclusion and exclusion criteria for the trial are shown in Fig S4. The clinical trial was separated into two phases: a water-only fasting period and a refeeding period (Fig S4). The trial lasted for 8 days: a 5-day period without any food intake followed by a 3-day interval to recover to normal food consumption. In brief, all the subjects fasted without any food, except unlimited mineral water. The trial subjects performed meditation and mild physical exercise during the 5-day fasting period. In refeeding period, 30%, 60% and 100% of total calorie intake was continuously arranged by the supervisors, which was on day 6, 7, and 8, respectively. Total calorie intake was calculated as the standard body weight in kilogram multiplied by 25 kcal according to the Guidelines for the Prevention and Treatment of Type 2 Diabetes in China (2020 Edition). For example, total energy intake is 1250 kcal for 50kg body weight. 30%, 60%, 100% of total energy equals to 375 kcal, 750 kcal, 1250 kcal on day 6, 7, and 8. Then, the required calorie is provided by rice flour (Shanghai Frog Rice Health Technology Co., Ltd, Shanghai, China), which contained 1647 kJ per 100 g (394kcal/100g). To meet the criterion of 30%, 60%, 100% of total energy, the person with 50kg body weight was given 95.17g, 190.36g and 317.26g rice flour on day 6, 7, and 8, respectively A 90-day follow-up period with ad-libitum food intake was monitored by periodic laboratory tests.

In order to ensure the patient’s compliance, three steps were taken: (1) the motivated and experienced participants were enrolled; (2) the participants were monitored by our clinical team; (3) A controlled environment was used during 5-day water-only fasting and 3-day refeeding.

**Assessment of physiological and** **laboratory indexes**

Waist circumference was measured at the midpoint between the rib and the iliac crest after inhalation and exhalation. Blood pressure was measured with an electronic blood pressure monitor (YuMell YE620A; YuMell Healthcare, China) on the right arm.

Laboratory assessments included complete blood-cell counts, plasma electrolytes, liver and renal function tests, fasting blood-glucose, insulin, IGF-1, and urine analysis, all performed at Longhua Hospital.

For evaluation of lymphocytes in peripheral blood, a routine clinical flow cytometry test protocol was used. Briefly, 100 μl whole blood was stained with appropriate antibodies in PBS containing 0.1% (wt/vol) BSA and 0.1% NaN_3_. The following antibodies were purchased from BD Biosciences (San Jose, CA, USA): anti-CD45(HI30), anti-CD3(SK7), anti-CD4 (RPA-T4), anti-CD8 (RPA-T8), anti-CD19(HIB19), anti-CD25 (M-A251), anti-CD127 (HIL-7R-M21). 1×FACS lysing solution (BD, 349202) 1 mL was added to remove red blood cells. After incubation of whole blood for 15 min at room temperature, cells were collected by centrifugation at 1200 rpm for 5 min and resuspended in FACS-staining buffer. The flow cytometry data were acquired on a BD FACSCelesta and analyzed with BD FACSDiva software.

**Questionnaire survey**

The questionnaire survey was conducted to record the personal details of the subjects, including their past medical history, marital and fertility history, life-style habits, previous experience with fasting, menstrual cycle for female subjects, ability to tolerate hunger, the Hamilton Depression Scale (HAMD) and Hamilton Anxiety Scale (HAMA).

**Metabolomic profiling of serum and urine samples**

Serum and urine samples were collected from all the participants the morning after 12 hours of water-only fasting. Serum samples were obtained by centrifugation at 3000 rpm for 10 min and immediately stored at -80°C until analysis. Urine samples were stored at -80 °C without any preservatives. A laboratory-developed test (LDT) kit –Metabolite Array (HMI, Shenzhen, Guangdong, China) was used to assess individual metabolites. The test was performed on an ultra-performance liquid-chromatography coupled to a tandem-mass-spectrometry (UPLC-MS/MS) system (HMI, Shenzhen, Guangdong, China). Sample preparation and derivatization protocols were based on previously-published methods [17]. All measurements were performed simultaneously at the end of the study to minimize variability.

**Statistical analysis**

Data are presented as mean ± standard deviation. Statistical analyses were performed using R studio, SPSS software, Excel and GraphPad Prism6 software (GraphPad Software, San Diego, CA, USA). For the comparison of continuous variables, data were compared with the baseline level via paired student’s t-test, but not corrected/adjusted for multiple testing. All statistical tests are 2-sided and P≤0.05 is considered statistically significant.

**Supplemental data**


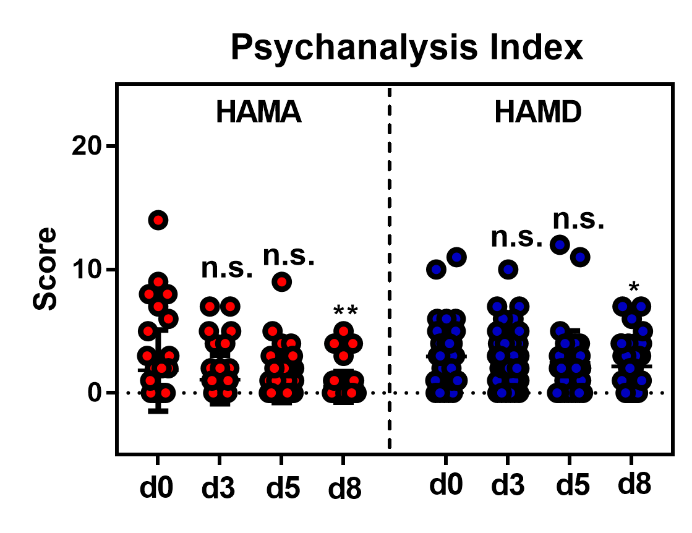


A

B


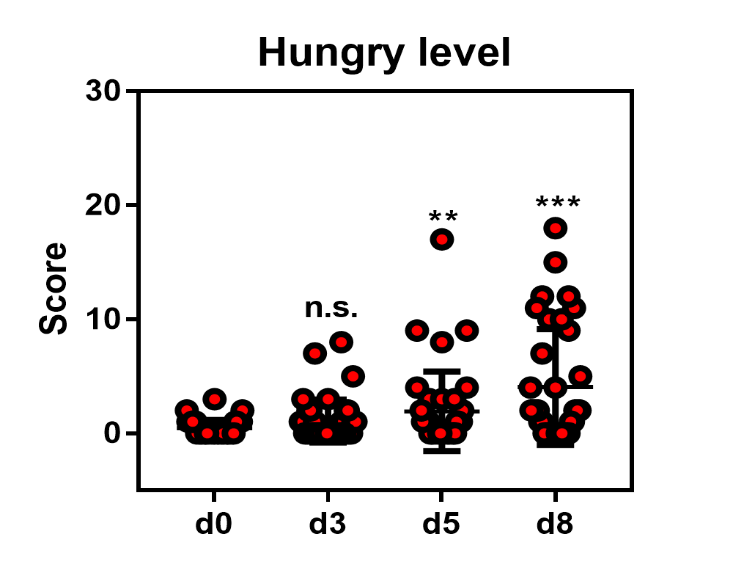
**Hunger level**

**Figure S1. Mental indexes associated with water-only fasting**

A. Hunger was increased upon water-only fasting. In the hunger questionnaire, a score of 20 represented extreme hunger, while a score of 0 represented no symptoms of hunger. B. Water-only fasting decreased the degree of anxiety and depression. The degree of anxiety and depression was determined by scales of anxiety (Hamilton Anxiety, HAMA) and depression (Hamilton Depression, HAMD). The degree of anxiety and depression was represented as higher scores for HAMA and HAMD.


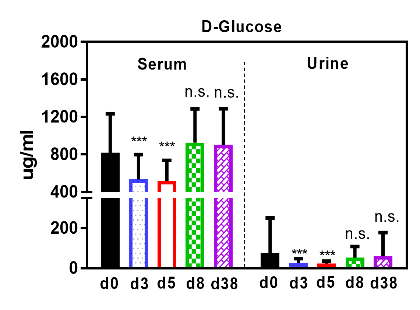


**D-Glucose**


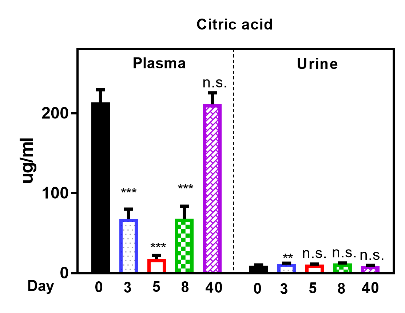

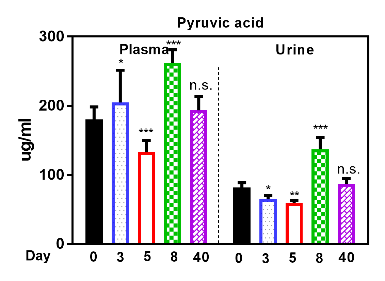

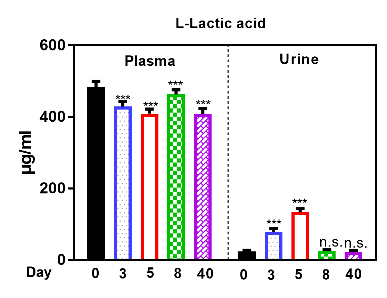

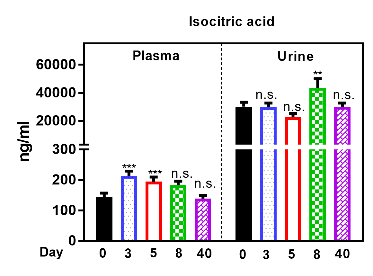

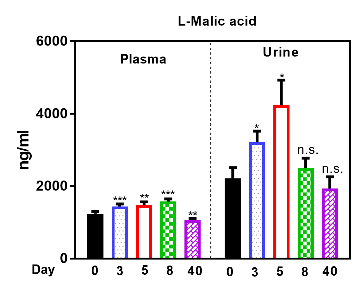


A

B

C

D

E

F

**Figure S2: Glycolysis was inhibited during water-only fasting**

A-F. A total of 6 metabolites involved in glucose metabolism were differentially expressed in both serum and urine, including glucose, pyruvate acid, isocitric acid, malic acid, citric acid and lactate acid during water-only fasting. Shown are average values with standard deviation (s.d.); * denotes P < 0.05, ** denotes P < 0.01 and ***denotes P < 0.001, n.s.= not significant


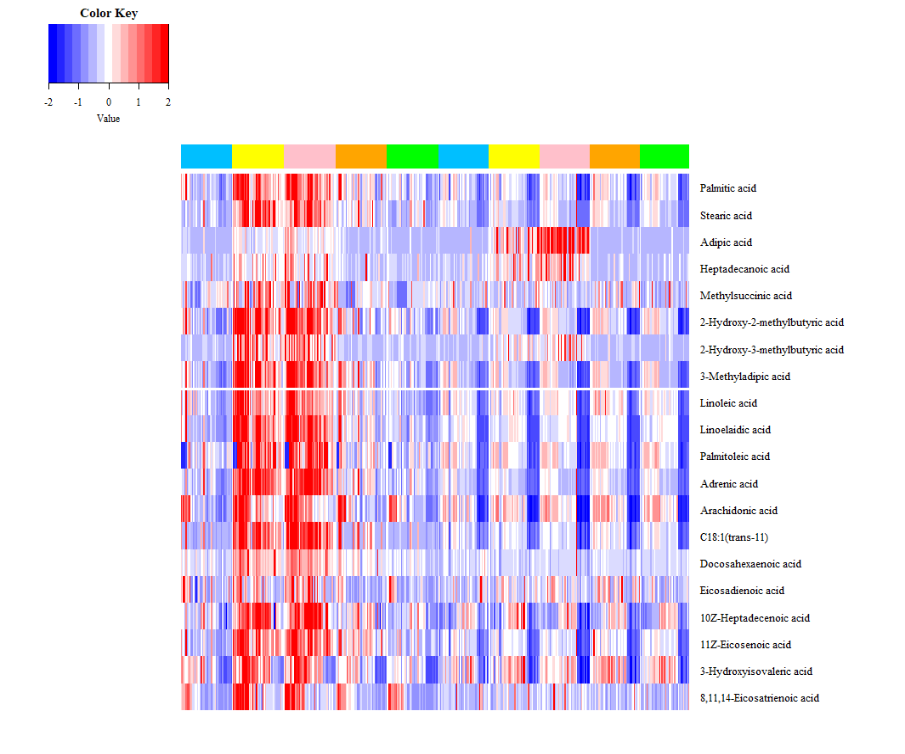


Saturated

fatty acid

Unsaturated

fatty acid

0d

3d

5d

8d

40d

Serum

Urine

0d

3d

5d

8d

40d

**Figure S3:** Heatmap of altered saturated fatty acids and unsaturated fatty acids during water-only fasting.


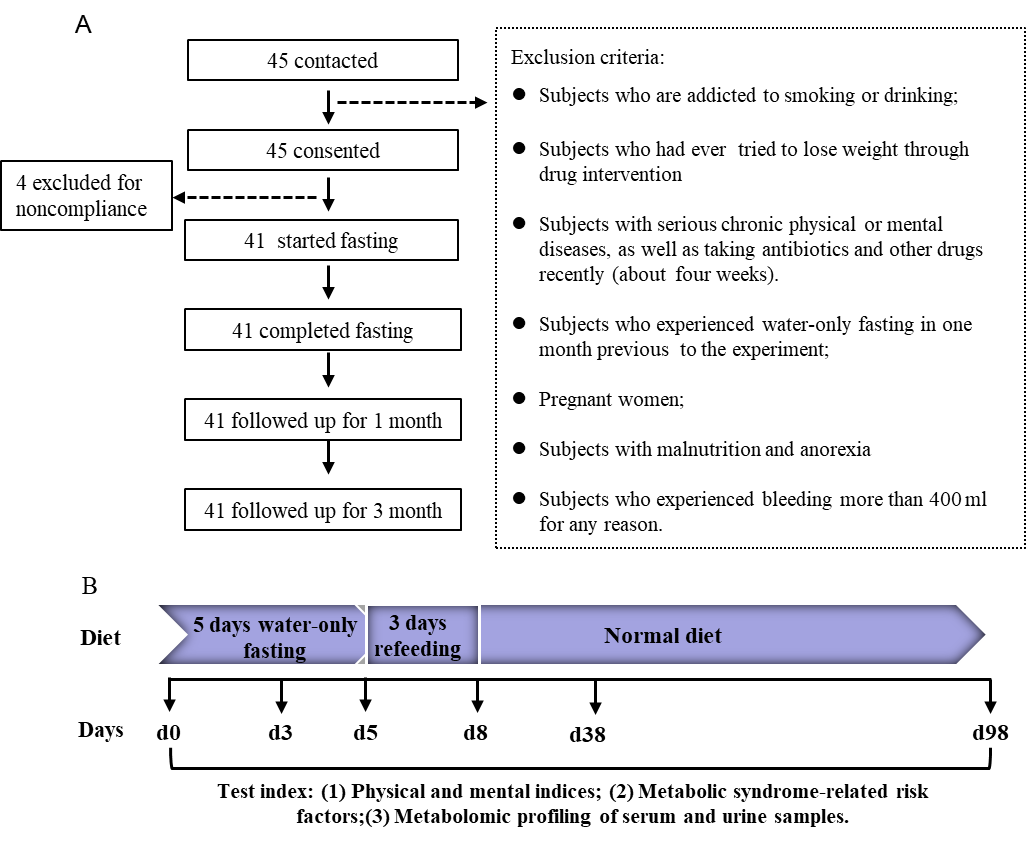


**Figure S4. Protocol of the water-only fasting study.**

A. Flow chart of the selection procedure for study subjects. B. Schedule of the fasting study. A total of forty-one participants who provided serum and urine samples at baseline (Day 0), fasting period (day 3 and day 5), 3 days (day 8) and 1 month post fasting (day 38), 3 months post fasting (day 98), were included in the present study.

**Table S1: Correlation of metabolic-syndrome -related risk makers to patient characteristics**

| Characteristics | Variable | BMI | Waist circumference | Diastolic BP | Systolic BP | FPG | Insulin | IGF-1 |
| --- | --- | --- | --- | --- | --- | --- | --- | --- |
| Gender | Male | -1.74 | -7.82 | -15.12 | -9.12 | -2.69 | -44.40 | -19.99 |
|  | Female | -1.58 | -11.08 | -11.88 | -11.67 | -2.26 | -36.35 | -60.24 |
|  | p Value | 0.2905 | 0.0026 | 0.3249 | 0.5134 | 0.2077 | 0.4470 | 0.0094 |
| Age | Age (>45) | -1.55 | -9.65 | -13.65 | -8.85 | -2.71 | -40.33 | -47.56 |
|  | Age (≤45) | -1.82 | -9.86 | -12.47 | -13.67 | -1.96 | -38.56 | -35.75 |
|  | p Value | 0.0836 | 0.8917 | 0.7260 | 0.2237 | 0.0273 | 0.8704 | 0.4737 |
| Number of previous Fasting | ≤5 | -1.66 | -9.07 | -11.59 | -9.41 | -2.39 | -45.38 | -33.11 |
|  | >5 | -1.64 | -10.20 | -14.38 | -11.46 | -2.47 | -35.65 | -49.82 |
|  | p Value | 0.9248 | 0.4455 | 0.3983 | 0.6001 | 0.8100 | 0.3573 | 0.3031 |

BMI: Body Mass index; DBP: Diastolic blood pressure; SBP: Systolic blood pressure; FPG: Fasting plasma glucose

**Table S2: Altered metabolites in serum and urine during water-only fasting**

| **Site** | **Total** | **Altered metabolites** | | | |
| --- | --- | --- | --- | --- | --- |
|  |  | **0d vs 3d** | **0d vs 5d** | **0d vs 8d** | **0d vs 40d** |
| Serum | 243 | 74  (47up, 27down) | 77  (50 up, 27 down) | 28  (16 up, 12 down) | 8  (2 up, 6 down) |
| Urine | 229 | 93  (62 up, 31 down) | 100  (37up, 63 down) | 62  (25 up, 37 down) | 17  (3 up, 14down) |

**Table S3. Baseline characteristics of water-only clinical trial participants**

| **Characteristics** | **(n = 41)** |
| --- | --- |
| **Sex, n (%)** | |
| Male | 17 (41.46) |
| Female | 24 (58.64) |
| **Age, n (%)** | |
| 26-35 | 5 (12.20) |
| 36-45 | 10 (24.39) |
| 46-55 | 16 (39.02) |
| 56-65 | 10 (24.39) |
| **Number of experienced Bigu, n (%)** | |
| 1-10 | 22 (53.66) |
| 11-20 | 15 (36.58) |
| 21-30 | 2 (4.88) |
| 31-40 | 1 (2.44) |
| >40 | 1 (2.44) |
| **General condition of subjects** |  |
| Body weight(kg) | 67.37 ± 13.60 |
| BMI (kg/m^2^) | 24.11±3.31 |
| Systolic pressure（mmHg) | 117.32±11.32 |
| Diastolic pressure (mmHg) | 77.12±9.44 |
| Heart rate(beats/min) | 69.07±7.34 |
| Waistline(cm) | 85.29±9.18 |
